# Validation of Three Different Sterilization Methods of Tilapia Skin Dressing: Impact on Microbiological Enumeration and Collagen Content

**DOI:** 10.3389/fvets.2020.597751

**Published:** 2020-12-23

**Authors:** Ahmed Ibrahim, Dalia Hassan, Noura Kelany, Saber Kotb, Mahmoud Soliman

**Affiliations:** ^1^Veterinary Teaching Hospital, Faculty of Veterinary Medicine, Assiut University, Assiut, Egypt; ^2^Department of Animal and Poultry Hygiene, and Environmental Sanitation, Faculty of Veterinary Medicine, Assiut University, Assiut, Egypt; ^3^Department of Veterinary Pathology and Clinical Pathology, Faculty of Veterinary Medicine, Assiut University, Assiut, Egypt; ^4^Laboratory of Veterinary Pathology, College of Veterinary Medicine, Chonnam National University, Gwangju, South Korea

**Keywords:** tilapia fish skin, biological dressing, sterilization, silver nanoparticles, collagen

## Abstract

Tilapia fish skin has demonstrated promise as a stable and practical biological dressing to be used in wound and burn management. However, the appropriate sterilization technique of the Tilapia fish skin is crucial before its clinical application. The standard sterilization technique must eliminate harmful pathogens but maintain the structural and biochemical properties that could compromise the dressing function. This study investigated and compared the efficiency of three sterilizing agents; chlorhexidine gluconate 4% (CHG), povidone iodine 10% (PVP-I), and silver nanoparticles (25 μg/mL) (AgNPs), at three different times (5, 10, and 15 min) on Tilapia fish skin based on the microbial count, histological and collagen properties. Among the sterilization procedures, AgNPs showed rapid and complete antimicrobial activity, with a 100% reduction in microbial growth of the fish skin throughout the treated times. Furthermore, AgNPs did not impair the cellular structure or collagen fibers content of the fish skin. However, CHG and PVP-I caused alterations in the collagen content. This study demonstrated that the AgNPs treatment of Tilapia fish skin provided sterile skin while preserving the histological properties and structural integrity. These findings provide an efficient and quick sterilization method suitable for Tilapia fish skin that could be adopted as a biological dressing.

## Introduction

Biological dressings, such as amnion, allografts, xenografts, bioengineered tissues, and cultured-wound healing cells, have been used recently for topical treatment of burns and skin wounds (1). Collagen dressings are widely used due to their beneficial properties including low antigenicity, enhanced inflammation and hemostasis, and accelerated fibroplasia and epithelization (2). Collagen dressings derived from cattle and pig skin or chicken waste are inappropriate due to the risk of disease transmission or religious and cultural reasons (3, 4). Marine fish skin is a good source of collagen, with antibacterial and antioxidant properties due to collagen peptides and omega-3 polyunsaturated fatty acids, particularly eicosapentaenoic acid (EPA) and docosahexaenoic acid (DHA). Thus, it is unlikely to be a source of infectious disease (3, 5–8).

The use of Nile Tilapia (*Oreochromis niloticus*) skin as a potential natural material in the management of burns and wounds arose because of its collagenous, histological, and mechanical comparability to human skin (9–11). Tilapia skin is a readily available, quality, safe, inexpensive material that is easy to apply (12). Nile tilapia skin also has a high level of biocompatibility in nature as the collagen extract is a biocompatible type I collagen with potential as a biomedical material for use in clinical regenerative medicine (13–16).

The sterilization of wound dressings before use on a patient is critical to guarantee the well-being of the patient and the healing process of the skin wound. Ordinary methods used to sterilize skin grafts are ethylene oxide gas sterilization, irradiation, and chemical sterilization (17–19). Ethylene oxide is a toxic gas which likewise generates harmful reaction products with potential hazards to the patient (20, 21). Sterilization of skin grafts with irradiation worsened the structure of the skin matrix since it can cause collagen matrix cross-linking or breakage of the peptide bonds within the collagen molecules (22). Unlike mammalian skin grafts, which require harsh chemical sterilization, Tilapia skin grafts require a gentle processing method because the colony forming units found in the tilapia skin samples suggested the existence of a normal, non-infectious microbiota (23). However, the fish skin may be contaminated with different microorganisms, such as *Pseudomonas aeruginosa, Aeromonas sobria, Klebsiella pneumonie, Enterococus faecalis, Aeromonas hydrophila, Proteus mirabilis, Globicatella sanguinis, Aeromonas veronii, Streptococcus uberis, Candida parapsilosis*, and *Streptococcus suis* from water environment or other sources of contamination (23, 24).

Chlorhexidine and povidone iodine are routinely used for sterilization and disinfection of grafting procedures as they are effective against Gram-negative, Gram-positive bacteria and fungi and kill by disruption of the cell membrane (17, 25–28). Silver nanoparticles have long been known as an antimicrobial agent and are used as silver-containing wound dressings and for disinfection of the wound graft (19, 29).

An effective sterilization method must eliminate or minimize microbial populations without changing the structure, bioactive composition, or biocompatibility of the skin grafts (17–19). To author's knowledge, there is a lack of studies on fish skin sterilization methods and effects on structure or function (17). The study purpose is to compare microbial count, collagen properties and histology of fish skin following three different sterilization times and methods.

## Materials and Methods

### Fish Skin Collection

Tilapia skin was collected from fresh Nile Tilapia (*Oreochromis niloticus*) fish (700 ± 40 gm, 22 ± 3 cm standard length), obtained from The Aquatic Medicine Unit's tanks, Faculty of Veterinary Medicine, Assiut University, Assiut, Egypt. The fish were euthanized physically by decapitation according to The American Association of Zoo Veterinarians (AAZV) Guidelines for euthanasia (30). Fresh skin samples were obtained by sharp and blunt dissection from the underlying muscles and washed with a sterile normal saline solution to remove any trace of blood and other impurities. The skin samples were then placed into a sterile normal saline solution and transected into strips 3 × 3 cm. Tilapia skin strips were immediately subjected to three different sterilization procedures (three skin samples in triplicate for each treatment), each at three contact times (5, 10, and 15 min) as described below.

### Sterilization Procedures

Sterilization procedures were carried out using three different sterilizing agents; the chlorhexidine gluconate 4% (CHG) (Hibiclens Mölnlycke Health Care, Norcross, GA), povidone iodine 10% (PVP-I) (BETADINE, El- Nile Co. for Pharmaceutical and Chemical Industries, Egypt), and silver nanoparticles (AgNPs) (25 μg/mL). The Tilapia skin strips were allocated randomly into four main groups (3 skin strips for each); CHG, PVP-I, and AgNPs groups. Each group was then incubated for different contact time points (5, 10, and 15 min). In addition to a control group (C) treated with normal saline for the three different times. The tilapia skin strips were subjected to bacteriological and histological evaluation following each sterilization treatment at the indicated time points.

### Preparation of Silver Nanoparticles

Stable silver nanoparticles (AgNPs) <100 nm were synthesized in a typical one-step protocol as described before (31). Briefly, 1.0 g of soluble starch was added to 100 mL of deionized water and heated until complete dissolution. One milliliter of 100 mM aqueous solution of silver nitrate (AgNO_3_) crystal (ACS AgNO_3_, F.W 169, 87 Gamma laboratory chemicals, Sigma-Aldrich, Germany, Assay: Min 99.0%) was added and stirred well. This mixture was put into a dark glass bottle and autoclaved. The resulting solution was clear yellow in color indicating the silver nanoparticles formation.

The stock solution of AgNPs was kept in dark glass bottles away from direct sunlight and at room temperature (25°C). The concentration and size of the particles were measured before using. The morphology and size of AgNPs were measured by transmission electron microscopy (TEM) (JEOL-JEM-100CX II) at the Electron Microscopy Unit, Assiut University (Supplementary Figure 1). The concentration of AgNPs stock was measured by Graphite Furnace Atomic Absorption Model 210VGP at the Faculty of Science, Assiut University.

### Microbiological Evaluation

Microbiological evaluation of the fish skin strips was performed according to APHA (32) and Qazi et al. (33). An area of 3 × 3 cm^2^ was aseptically swabbed for microbial count. Each of Plate count agar “HMESIA- Ref -M091A,” Manitol Salt Agar “Oxoid CM0085,” MacConkey Agar OMRI W/O Crystal violet “Biolife “and Difco^TM^ Potato Dextrose Agar- Ref 213400 were used for Total viable bacteria, Staphylococci, Coliforms, Yeasts and Molds count, respectively. The Pour plate method was used for the enumeration of colony-forming units (CFU/cm^2^) on Plate Count Agar. The swab was transferred immediately into 10 ml sterile nutrient broth. One milliliter from the broth was transferred aseptically into a sterilized petri-dish in triplicate. Ten to fifteen milliliter of the dissolved Plate count agar media was cooled in water bath to a temperature of 44–46°C and poured aseptically into each plate. The dish was moved in a circular motion several times to ensure homogeneity of the sample with the medium. The plate was left until the hardening of the medium, and then incubated upside down in the incubator at a temperature of 37°C for 24 h. After incubation period the total number of each replicate of the sample was counted and the mean extracted and multiplied by the inverse of dilution. The resulting colonies expressed as CFU/ml. The same technique was used for Staphylococci, Coliforms, Yeasts and Molds count. Staphylococcal count was plated on Manitol Salt Agar “Oxoid CM0085” at 37°C for 24 h, Coliform count on MacConkey Agar OMRI W/O Crystal violet “Biolife at 37°C for 18–24 h, and Yeasts and Molds was plated on Difco^TM^ Potato Dextrose Agar- Ref 213400 incubated at 28 ± 2°C for 24–72 h.

### Sterilization Efficiency

The efficiency of each sterilization procedure was calculated by comparing the bacterial count before and after each treatment for each contact time via the following equation:

Disinfection efficacy % = (C_0_ – C)/C_0_ × 100, where (C_0_) is the initial bacterial count (control negative) and (C) is the count of bacteria after a certain contact time of the sterilizing agent (34).

### Histological Evaluation

Specimens (0.5 × 0.5 cm) of the Tilapia skin strips were collected from the groups following each sterilization treatment at different times and fixed in 10% neutral buffered formalin. The formalin-fixed skin samples were routinely processed and embedded in paraffin. They were then sectioned at 5 μm thickness and stained with Mayer's hematoxylin (Merck, Darmstadt, Germany) and eosin (Sigma, Missouri, USA). Afterwards, the slides were examined microscopically and the histological evaluation was performed in a blind fashion on coded samples, and a comparison was made with the sections from the control-treated group. Histological evaluation to assess integrity and organization of collagen fibers was based on 0–3 scale (35, 36). Collagen integrity scores: 0 = continue, long fiber, 1 = slightly fragmented, 2 = moderately fragmented, 3 = severely fragmented. Collagen organization scores: 0 = compact and parallel, 1 = slightly loose and wave, 2 = moderately loose, wavy and cross to each other, 3 = no identifiable pattern.

### Histochemical Staining of Collagen

Sirius red stain was used for evaluation of the collagen content (37). Paraffin-embedded sections were deparaffinized in xylene and rehydrated in a graded series of ethanol solutions [into 0.1 M phosphate-buffered saline (PBS), pH 7.2] to distilled water. The sections were then stained with Sirius red stain following the manufacturer's protocol, dehydrated in graded alcohol, made transparent with xylene, and mounted. Afterwards, slides were observed under the microscope to check for collagen staining with red color. The collagen content was evaluated according to the depth of the red color.

Using threshold area fraction determination, the percentage of collagen positive area was calculated using ImageJ (17). The amount of collagen was reported as a percentage from the total number of pixels in the optical view as a percentage and expressed as mean ± SEM.

### Negative Image Study Using CMEIAS Color Segmentation

An improved computing technology has been used to process color images by segmenting background object pixels from the foreground object (38, 39), making this technique especially useful due to the complex color that existed for this analysis.

### Statistical Analyses and Software

Statistical analyses were performed by one-way ANOVA using GraphPad Prism software version 5.03 (GraphPad Software Inc., La Jolla, CA, USA). *P*-values of < 0.05 were considered statistically significant. Figures were generated using Adobe Photoshop CS6 and Prism 5 version 5.03.

## Results

### Microbiological Evaluation of the Treated Skin

The effectiveness of chlorhexidine gluconate 4%, povidone iodine 10%, and silver nanoparticles (25 μg/mL) on the microbial count of the fish skin at the different three contact time points was summarized in Table 1. The silver nanoparticles (AgNPs) achieved a 100% reduction percentage against aerobic bacterial, Staphylococcal, Coliform, and Yeast and Mold counts throughout the treatment time points (5, 10, 15 min). However, povidone-iodine achieved a 100% reduction against the Coliform and Yeast and Mold counts at the contact time of 10 min, and against the aerobic bacterial and Staphylococcal counts at the contact time of 15 min. Chlorhexidine gluconate achieved 100% reduction against Staphylococcal and Yeast and Mold counts after the treatment time of 15 min.

**Table 1:** Mean value of microbial count before and after application of antiseptics.

**Treatment**	**Time (min)**	**Microbial counts of fish skin CFU/cm**^****2****^ **(Mean value** **±** **S.E)**							
		**Aerobic bacterial count**		**Staphylococcal count**		**Coliform count**		**Yeast and mold count**	
			**Reduction %**		**Reduction %**		**Reduction %**		**Reduction %**
Control		5 × 10^4^ ± 1.32^a^		1.8 × 10^4^ ± 0.8^ab^		2.2 × 10^4^ ± 1.79^abc^		1.2 × 10^4^ ± 1.29 ^abd^	
Chlorhexidine gluconate (CHG)	5	2.4 × 10^4^ ± 0.92^b^	52%	7 × 10^3^ ± 1.86^bc^	61.11%	1 × 10^4^ ± 0.45^bac^	54.54%	7 × 10^3^ ± 0.11^abf^	41.66%
	10	6 × 10^3^ ± 1.54^c^	88%	1 × 10^3^ ± 1.02^ac^	94.44%	2 × 10^3^ ± 2.32^bca^	90.90%	2 × 10^3^ ± 1.92^afb^	83.33%
	15	0.1 × 10^3^ ± 1.82^c^	99.80%	0.0 ± 0.0^ac^	100%	0.1 × 10^3^ ± 0.82^bca^	99.54%	0.0 ± 0.0^afb^	100%
Povidone- iodine (PVP-I)	5	1 × 10^4^ ± 0.92^d^	80%	6 × 10^3^ ± 1. 87^ad^	66.66%	4 × 10^3^ ± 1.02^adf^	81.81%	2 × 10^3^ ± 1.02^adi^	83.33%
	10	1 × 10^3^ ± 0.52^e^	98%	1 × 10^3^ ± 0.98^ade^	94.44%	0.0 ± 0.0^adg^	100%	0.0 ± 0.0^aid^	100%
	15	0.0 ± 0.0^e^	100%	0.0 ± 0.0^ade^	100%	0.0 ± 0.0^adg^	100%	0.0 ± 0.0^aid^	100%
Silver nanoparticles (AgNPs)	5	0.0 ± 0.0^f^	100%	0.0 ± 0.0^adc^	100%	0.0 ± 0.0^adh^	100%	0.0 ± 0.0^abi^	100%
	10	0.0 ± 0.0^f^	100%	0.0 ± 0.0^adc^	100%	0.0 ± 0.0^adh^	100%	0.0 ± 0.0^abi^	100%
	15	0.0 ± 0.0^f^	100%	0.0 ± 0.0^adc^	100%	0.0 ± 0.0^adh^	100%	0.0 ± 0.0^abi^	100%

*a–i, ab, bc, ac, ad, ade, adc, abc, bac, bca, adf, adg, adh, abd, abf, afb, adi, aid, and abi values with no common superscripts differ significantly (P < 0.05)*.

### Histological Evaluation of the Treated Fish Skin

**Figure 1:**
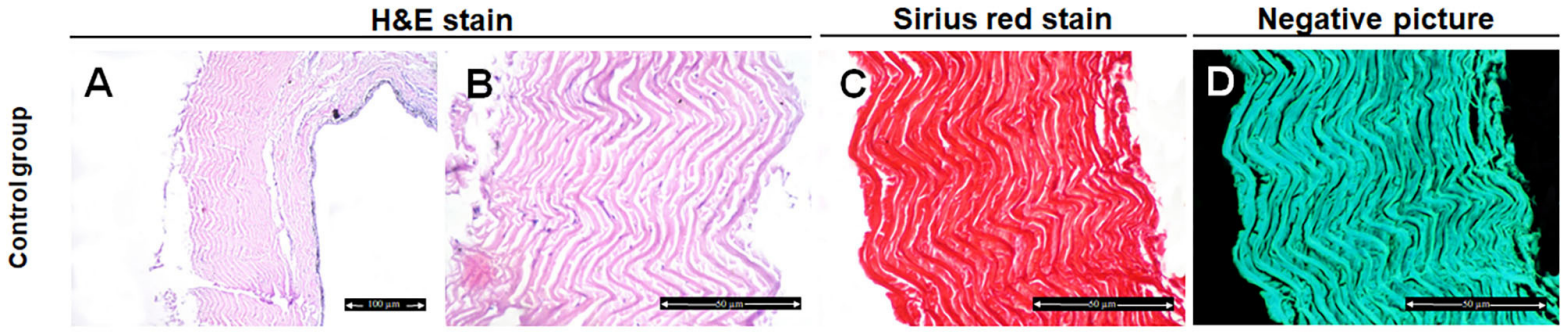
Histological and histochemical evaluation of Tilapia fish skin in the control group (treated with normal saline). **(A,B)** Hematoxylin and eosin stained sections. **(C)** Sirius red stained sections for collagen. **(D)** Negative image for **(C)** using CMEIAS Color Segmentation. The scale bars in **(A)** = 100 μm and **(B–D)** = 50 μm.

Histological analysis of the fish skin samples from the control group showed compactly arranged bundles of collagen fibers in the dermis and occasionally focal epithelial coating and superficial melanophores were seen. The collagen fibers were well organized and distributed in a parallel pattern with no evidence of disaggregation (Figures 1A,B).

**Figure 2:**
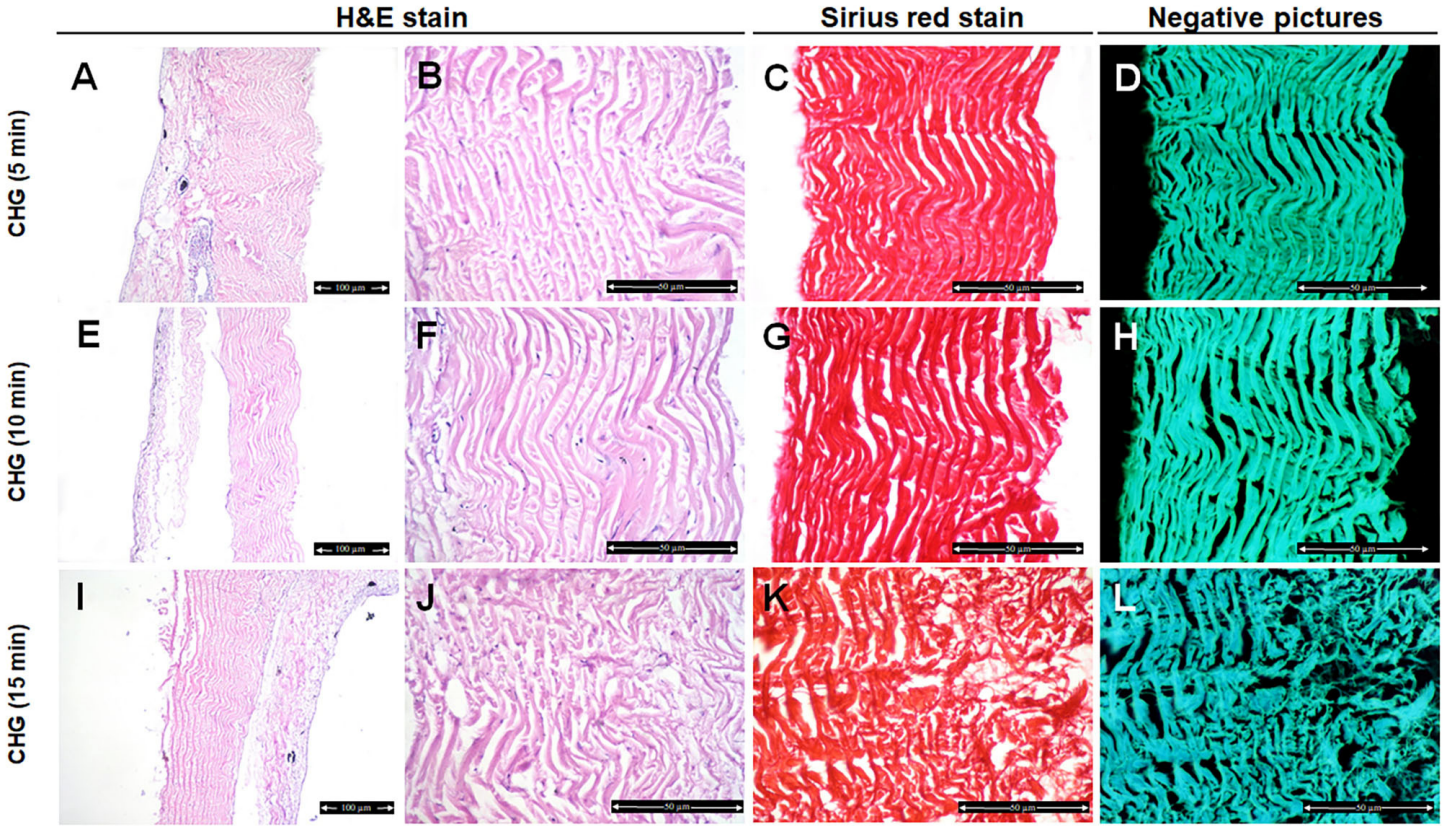
Histological and histochemical evaluation of Tilapia fish skin in the chlorhexidine gluconate (CHG) group. Hematoxylin and eosin stained sections treated with CHG for 5 min **(A,B)**, 10 min **(E,F)**, and 15 min **(I,J)**. **(C,G,K)** Sirius red stained sections for collagen. **(D,H,I)** Negative images for **(C,G,K)**, respectively, using CMEIAS Color Segmentation. The scale bars in (**A,E,I)** = 100 μm and **(B–D,F–H,J–L)** = 50 μm.

Fish skin treated with CHG for 5 min showed well organized and parallel collagen fibers (Figures 2A,B) with mild changes in the collagen fibers after 10 min treatment (Figures 2E,F). However, disorganized and disaggregated collagen fibers were obviously seen after 15 min treatment (Figures 2I,J,5) (*P* < 0.0001).

**Figure 3:**
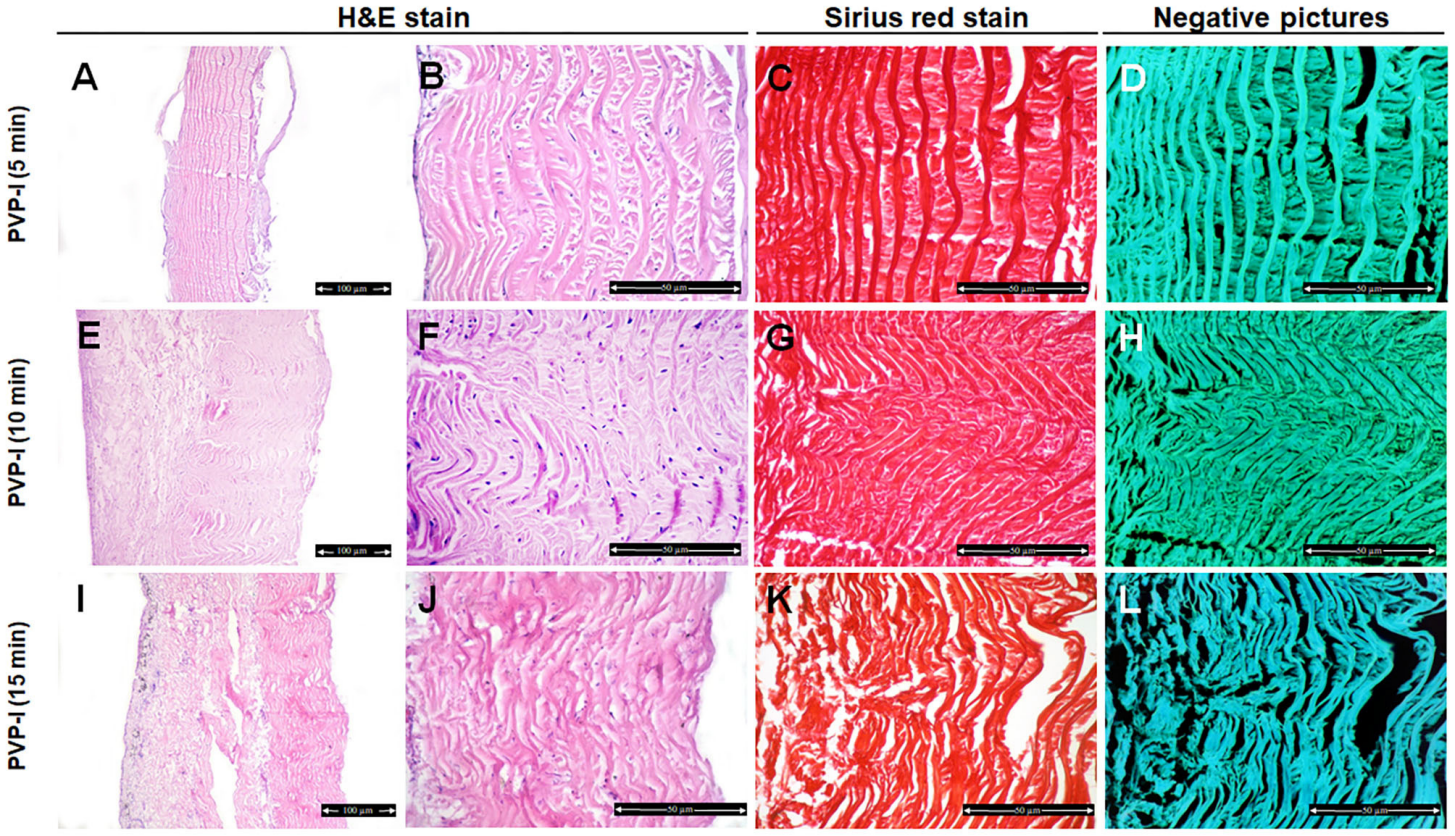
Histological and histochemical evaluation of Tilapia fish skin in the povidone iodine (PVP-I) group. Hematoxylin and eosin stained sections treated with PVP-I for 5 min **(A,B)**, 10 min **(E,F)**, and 15 min **(I,J)**. **(C,G,K)** Sirius red stained sections for collagen. **(D,H,I)** Negative images for **(C,G,K)**, respectively, using CMEIAS Color Segmentation. The scale bars in **(A,E,I)** = 100 μm and **(B–D,F–H**, **J–L)** = 50 μm.

In the PVP-I-treated group, the structure of the collagen fibers has not been changed after 5 min treatment (Figures 3A,B). Disorganization and disaggregation of the collagen fibers were observed after 10 min and 15 min treatment and were more prominent at 15 min treatment (Figures 3E,F,I,J,5) (*P* < 0.0001).

**Figure 4:**
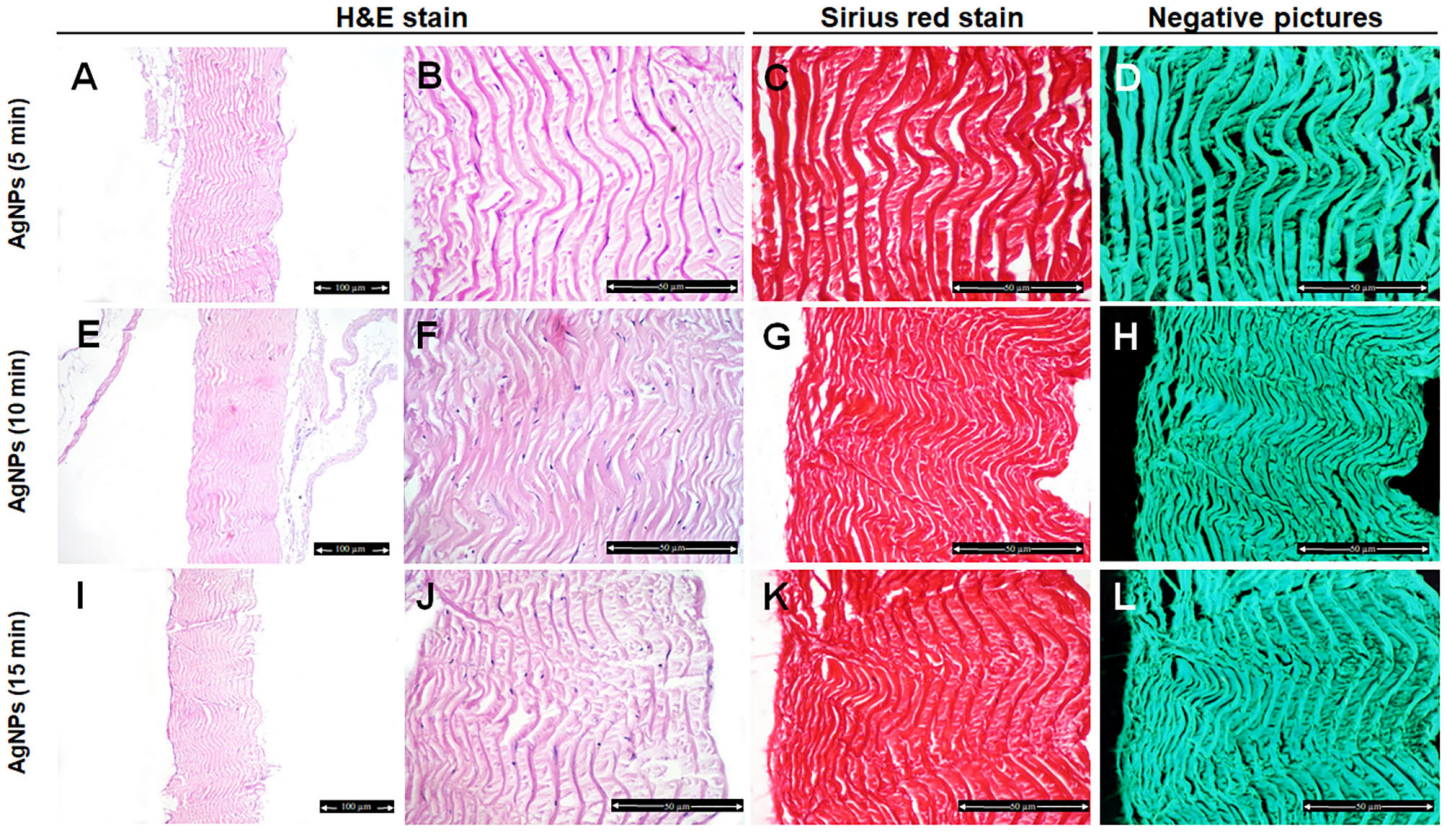
Histological and histochemical evaluation of Tilapia fish skin in the silver nanoparticles (AgNPs) (25 μg/mL) group. Hematoxylin and eosin stained sections treated with AgNPs for 5 min **(A,B)**, 10 min **(E,F)**, and 15 min **(I,J)**. **(C,G,K)** Sirius red stained sections for collagen. **(D,H,I)** Negative images for **(C,G,K)**, respectively, using CMEIAS Color Segmentation. The scale bars in **(A,E,I)** = 100 μm and **(B–D,F–H,J–L)** = 50 μm.

However, there was no change in the organization pattern of disaggregation of the collagen fibers in the AgNPs-treated group after 5–15 min treatment in comparing with control samples (Figures 4A,B,E,F,I,J).

### Histochemical Evaluation of the Treated Fish Skin

**Figure 5:**
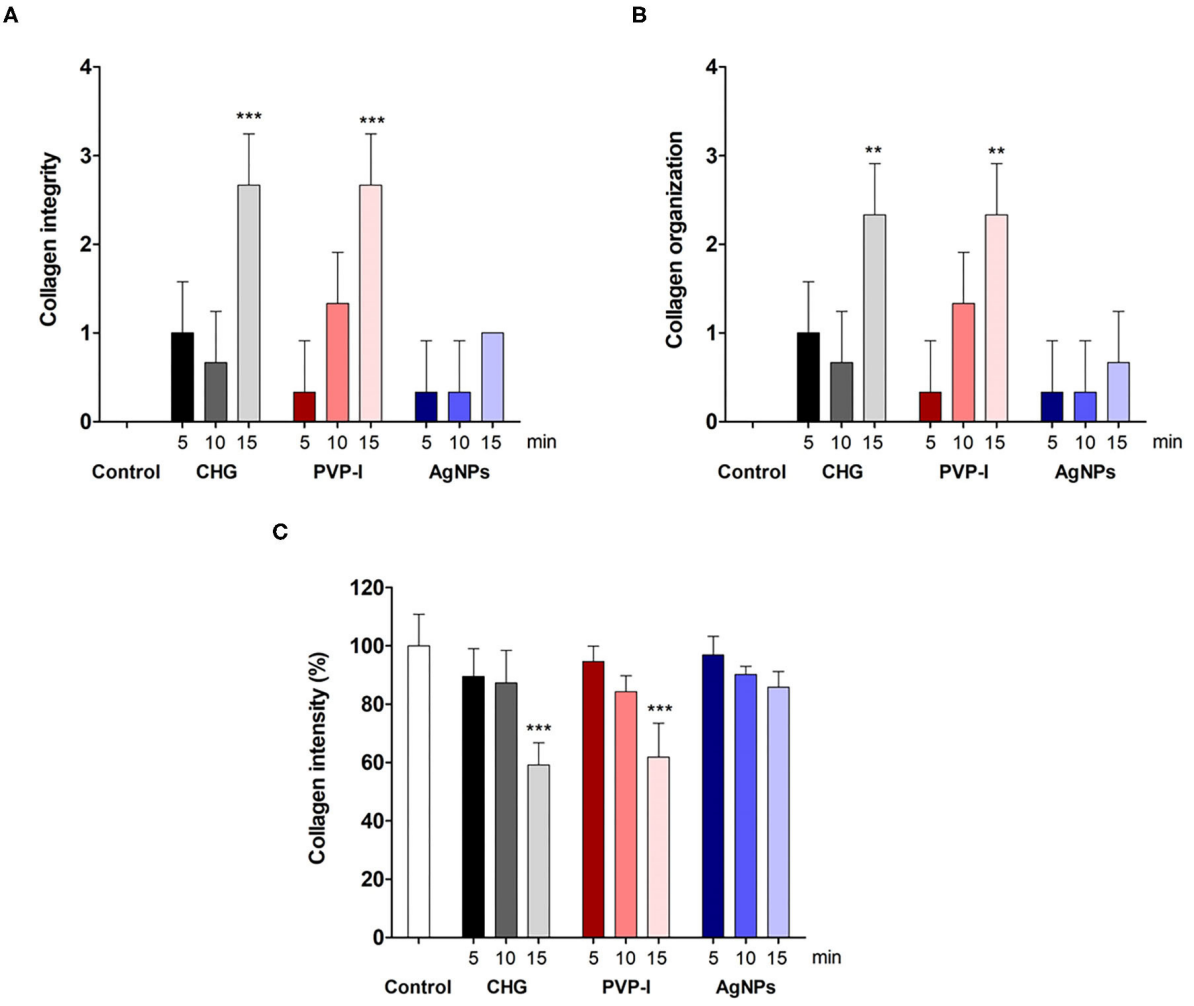
Evaluation of the collagen integrity, organization, and intensity in Tilapia fish skin under various sterilization methods. Collagen integrity **(A)** and organization **(B)** were evaluated based on 0–3 scale as described in the materials and methods. The collagen intensity **(C)** was quantified using ImageJ. The results are expressed as a percentage from the total number of pixels, and are normalized to control-treated group. Differences were evaluated using one-way ANOVA. **p < 0.001; ***p < 0.0001.

We further examined the organization of the collagen fibers by staining the skin samples from the control and treated groups with Sirius red stain and measured the collagen intensity in the fish skin using ImageJ. The fish skin treated with CHG for 5 min (Figures 2C,D) or PVP-I for 5 min (Figures 3C,D) showed well-organized of the collagen fibers and the preservation of the collagen intensity compared to control samples (Figures 1C,D). There was mild change in the disposition pattern of collagen fibers and the collagen intensity after sterilization for 10 min with CHG (Figures 2G,H, 5) or PVP-I (Figures 3G,H,5). However, the reduction in intensity of the collagen in the fish skin was statistically significant (*P* < 0.0001) after sterilization for 15 min with CHG or PVP-I (Figures 2K,L,3K,L,5). On the other hand, the sterilization of the fish skin with AgNPs showed well organized collagen fibers (Figures 4C,D,G,H,K,L) with non-significant change in the intensity of the collagen (Figure 5), regardless of the treated time.

## Discussion

Use of Tilapia skin xenografts as a biologic dressing for wound management is expanding (1). The fish skin is characterized by large amounts of moisture and collagen proteins at levels comparable to human skin. This prevents scarring while promoting the healing of wounds. In addition, unlike the gauze bandages, the tilapia skin does not need to be changed every day (3, 5, 6, 9–11).

Critical to fish skin use is applying a sterilization process with minimal impact on its positive qualities, especially with respect to zoonotic disease. The zoonotic diseases associated with fish contact are primarily bacterial infections. Most persons have been infected via an existing wound or fresh puncture wound while handling live or dead fish. These include *Mycobacterium, Erysipelothrix, Campylobacter, Aeromonas, Vibrio, Edwardsiella, Escherichia, Salmonella, Klebsiella*, and *Streptococcus iniae* (40–43). Therefore, the present study compared three different sterilization procedures; chlorhexidine gluconate (CHG), povidone iodine (PVP-I), and silver nanoparticles (AgNPs). Our results indicated that AgNPs are more efficient in reducing the microbial counts and preserving the structural components of the fish skin, thereby fulfilling the ideal sterilization agent requirement.

CHG has been used to disinfect the skin before surgery and sterilize surgical instruments and biological dressings (17, 28, 44). Consistent with previous data (17), our results showed that 4% CHG was effective in the reduction of microbial load of tilapia fish skin in variable degrees and the bactericidal effect increased with increasing contact time. CHG is a bisbiguanide compound that has a broad spectrum of activity against gram-positive and gram-negative bacteria and fungi, even in the presence of interfering materials, such as blood or serum (25, 44). It dissociates and releases the positively charged chlorhexidine cation. In turn, this cation binds to the negatively charged bacterial cell walls resulting in leakage of cellular contents, precipitation of intracellular compounds, and inhibition of adenosine triphosphate, which eventually inactivate or kill the bacteria (25, 44). In this study, complete inhibition of microbial contamination of fish skin was not achieved even with increasing the contact time till 15 min. This may be attributed to the fact that CHG is a more potent antiseptic against gram-positive than gram-negative bacteria and the development of resistance to gram-negative bacteria such as *Pseudomonas* spp. (45, 46).

On the other hand, the arrangement and content of the collagen fibers were not affected after CHG treatment for 5 min. However, long exposure to CHG for 10 or 15 min resulted in dissociation and disintegration of collagen fibers with decrease in the collagen intensity. Our results are in accordance with that reported by Alomar et al. (25) and are contradictory to Alves et al. (17).

PVP-I is routinely used as an antiseptic agent with a broad spectrum activity against bacteria, fungi, and yeast. It is a complex of polyvinylpyrrolidone iodine that gradually releases iodine as a free iodine, which provides antimicrobial efficacy through lipid iodination and oxidation of cytoplasmic and membrane compounds. Additionally, polyvinylpyrrolidone has a high affinity for cell membranes, supplying free iodine directly to the target cell and contributing to rapid microbial kill (44, 47, 48).

The current study demonstrated that fish skin treated with PVP-I had significantly reduced microbial count up to 100% by increasing the contact time up to 15 min. These findings were in accordance with Mann-Salinas et al. (28). Although PVP-I has a minimal toxic potential compared to CHG, it also induced alterations in the collagen fibers (dissociation and disintegration of collagen fibers) of the tilapia fish skin treated with PVP-I for 10 or 15 min, while, these changes were not noted in skin samples submitted for 5 min treatment.

Silver has been widely used in chronic wounds and burns care. Moreover, a number of silver-impregnated dressings have been developed (49–52). Silver has broad spectrum antimicrobial properties against a wide spectrum of gram-positive and gram-negative bacteria. It binds to negatively charged components in proteins and nucleic acids, thereby effecting structural changes in bacterial cell walls, membranes, and nucleic acids that affect viability. It is believed that it inhibits proteins by binding to and denaturing their thiol groups and preventing DNA replication by its condensation (49–52). Therefore, silver impregnation of biological-derived wound dressings has been recently used and it has been reported that silver impregnation of amniotic membranes combats microbial infection without any change on the physical characteristics (19, 29).

Interestingly, the microbial growth was rapidly and completely vanished by using AgNPs as an antiseptic for fish skin at all used treatment times (5–15 min) compared to CHG or PVP-I. Furthermore, the rapid antimicrobial activity of AgNPs is an obvious option so that the operative time is not extended beyond the minimum necessary.

By controlling the bioburden, AgNPs facilitate less toxic metabolites production, which result in a better collagen fibers preservation in the Tilapia skin. Furthermore, eukaryotic cells are less prone to silver toxicity than prokaryotes, which is thought due to an increased degree of structural and functional stability in eukaryotes (53). However, it should be noted that this study has a potential limitation, the storage of the fish skin after processing and sterilization. Therefore, further studies should address the storage and preservation of the sterilized fish skin samples.

## Conclusion

Sterilization of Tilapia fish skin, used as a biological dressing, using AgNPs is effective with a 100% reduction against microbial growth and without any change in the collagen content at three different times (5, 10, and 15 min). This is the first study to report the excellent performance of AgNPs as a quick and efficient sterilizer procedure for the Tilapia fish skin, relevant for clinical use as a biological dressing.

## Data Availability Statement

The raw data supporting the conclusions of this article will be made available by the authors, without undue reservation.

## Ethics Statement

The animal study was reviewed and approved by National Ethical Committee of the Faculty of Veterinary Medicine, Assiut University, Assiut, Egypt.

## Author Contributions

AI, SK, and MS designed the study, helped data interpretation, discussed the results, and edited the manuscript. AI, SK, NK, and MS performed the experiments. AI, SK, DH, and MS analyzed the data. MS wrote the manuscript. All authors contributed to the article and approved the submitted version.

## Conflict of Interest

The authors declare that the research was conducted in the absence of any commercial or financial relationships that could be construed as a potential conflict of interest.

## Supplementary Material

Supplementary Figure 1 Transmission Electron Microscopy (TEM) micrograph of silver nanoparticles showing spherical-shaped particles ranging from 8 to 35 nm (scale bar = 100 nm).
