# Supplementary figures and images for: Validation of Three Different Sterilization Methods of Tilapia Skin Dressing: Impact on Microbiological Enumeration and Collagen Content
Source: Front Vet Sci. 2020 Dec 23;7:597751. doi: 10.3389/fvets.2020.597751 (PMC7785820; doi:10.3389/fvets.2020.597751)

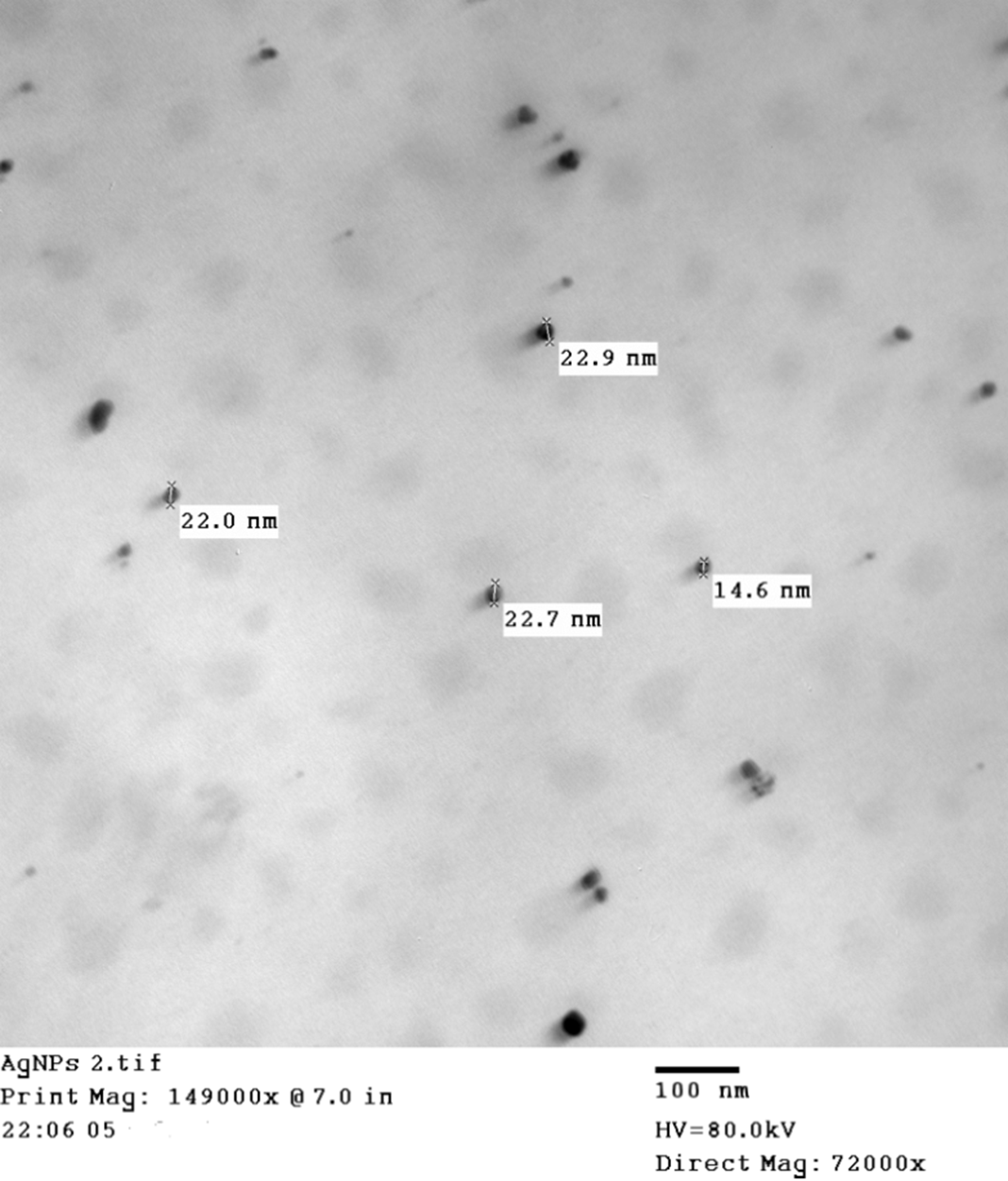

Supplement: Supplementary Figure 1 — Transmission Electron Microscopy (TEM) micrograph of silver nanoparticles showing spherical-shaped particles ranging from 8 to 35 nm (scale bar = 100 nm). [file Image_1.TIF]
